# 免疫检查点抑制剂相关神经系统不良反应的临床诊治建议

**DOI:** 10.3779/j.issn.1009-3419.2019.10.05

**Published:** 2019-10-20

**Authors:** 佳宇 史, 婧雯 牛, 东超 沈, 亦 李, 明生 刘, 颖 谭, 丽英 崔, 宇宙 管, 力 张

**Affiliations:** 1 100730 北京，中国医学科学院，北京协和医院神经科 Departmet of Neurology, Peking Union Medical College Hospital, Peking Union Medical College and Chinese Academy of Medical Sciences, Beijing 100730, China; 2 100730 北京，中国医学科学院，北京协和医院呼吸科 Department of Respiratory Medicine, Peking Union Medical College Hospital, Peking Union Medical College and Chinese Academy of Medical Sciences, Beijing 100730, China

**Keywords:** 免疫检查点抑制剂, 神经系统不良反应, 糖皮质激素, Immune checkpoint inhibitor, Nervous system, Steroids

## Abstract

免疫检查点抑制剂（immune checkpoint inhibitors, ICIs）可引起神经系统的不良反应，发生率在0.1%-12%，80%发生在应用前4个月内。可以引起神经系统各部位的病变，包括无菌性脑膜炎，脑膜脑炎，坏死性脑炎，脑干脑炎，横贯性脊髓炎等中枢神经系统病变，也可引起颅神经周围神经病、多灶性神经根神经病、格林巴利综合征、脊神经根神经病、重症肌无力、肌病等周围神经病变。对于神经系统的这些并发症，均需要症状体征，并结合影像学、脑脊液细胞学、脑电图或肌电图等检查，除外感染或恶性肿瘤后获得诊断。治疗中，在严重病例需停用ICIs，并用大剂量糖皮质激素或丙种球蛋白治疗和对症支持治疗。在出现神经系统不良反应后，严重病例预后较差。

## Abstract (en)

Immune checkpoint inhibitors (ICIs) can cause adverse reactions in the nervous system, with the incidence rate ranging from 0.1% to 12%, and 80% occurring within the first 4 months of ICI application. It can cause lesions in various parts of the nervous system, including aseptic meningitis, meningoencephalitis, necrotizing encephalitis, brainstem encephalitis, transverse myelitis and other central nervous system diseases. It can also cause cranial peripheral neuropathy, multifocal radicular neuropathy, Guillain-Barre syndrome, spinal radicular neuropathy and myasthenia gravis, myopathy, etc. For these complications of the nervous system, diagnosis could be made by sufficient collection of disease manifestations combined with imaging, cerebrospinal fluid examinations, electro-encephalogram or electro myography to exclude infection or tumor progression. In the treatment of severe cases, ICIs should be discontinued and treated with high doses of glucocorticoid or gamma globulin with systemic support. After neurological adverse reactions, the prognosis of severe cases is poor.

免疫检查点抑制剂（immune checkpoint inhibitors, ICIs）是针对程序性死亡受体1（programmed cell death protein 1, PD-1）抗体和细胞毒性T淋巴细胞相关抗原4（cytotoxic T lymphocyte associated antigen-4, CTLA-4）抗体，针对免疫T细胞激活过程中的两个关键通路：CTLA-4/B7-1/2和PD-1/对程序性死亡受体配体1（programmed cell death protein ligand 1, PD-L1），使肿瘤相关抗原无法启动活化信号通路而对多种肿瘤有抑制作用。迄今为止，美国食品和药物管理局（Food and Drug Administration, FDA）共计批准了6种ICIs，包括：O药Opdivo（nivolumab，纳武利尤单抗，欧迪沃）；K药Keytruda（pembrolizumab，帕博利珠单抗，可瑞达）；T药Tecentriq（Atezolizumab，阿特珠单抗，特善奇）；I药Imfinzi（Duravalumab，度伐单抗）；B药Bavencio（Avelumab，阿维鲁单抗）；L药Libtayo（Cemiplimab-rwlc，依匹单抗）治疗从膀胱癌到头颈癌和晚期非小细胞肺癌的各种类型。随着此类药物上市后应用的扩展，药物相关性不良反应也见诸多报道。ICIs带来的免疫治疗特有的免疫相关不良反应，称为免疫相关的不良事件（immune-related adverse effects, irAEs）。本文对神经系统irAEs发生率、临床表现、诊断和处理做一概述，提高对神经专科和肿瘤专科医生的认知和预警能力以及治疗能力。

## 1 神经系统不良反应的发生率

关于ICIs的神经系统不良反应发生率的报道相对较少，其中一项包括59项临床研究、涉及920例患者的回顾性调查分析表明，CTLA-4抑制剂致神经系统irAEs的发生率为3.8%，PD-1抑制剂为6.1%，CTLA-4抑制剂联合PD-1抑制剂为12.0%^[1]^。Ⅲ期临床实验（EORTC18071）报道成人抗CTLA-4（易普利姆单抗）组的神经系统irAEs的发生率为4%^[2]^。严重（3级-4级）神经系统irAEs发生在1.9%的抗CTLA-4（易普利姆单抗）患者中，0.2%-0.4%的抗PD-1和0.1%-1%的抗PD-L1患者中^[3]^。Cuzzubbo等^[1]^对27例接受ICI治疗并发生神经系统irAEs的患者进行分析，结果提示神经系统irAEs发生的中位时间为6周（1周-74周），所有病例的发生均是急性或亚急性的，并且与肿瘤应答反应相关。Spain等^[4]^与Zimmer等^[5]^两项研究报道表明，接受ICIs治疗发生irAEs患者中，分别有80%和75%的患者发生于开始接受免疫疗法的前4个月内。由此可见，神经系统的irAEs多发生于患者ICIs治疗的诱导阶段，建议重点监测前4个月内神经系统irAEs的发生。基于以上研究结果，神经系统irAEs为ICIs常见（≥1%）的不良反应，应予以重视。已有文献报道的神经系统相关irAEs总结见表 1。

**1:** 神经系统irAEs总结 Summary of nervous systemirAEs

ICIs	Central nervous system irAEs	Peripheral nervous system irAEs
Anti CTLA-4 Ipilimumab	Transverse myelitis Aseptic meningitis Meningoencephalitis Necrotizing encephalitis Brainstem encephalitis	Pericranial neuropathy Polyradiculoneuropathy Guillain-Barre syndrome Spinal radiculopathy myasthenia gravis
Anti PD-1	Limbic encephalitis Inflammatory demyelinating disease of central nervous system Brainstem encephalitis	Guillain-Barre syndrome myasthenia gravis
ICIs: immune checkpoint inhibitors; PD-1: programmed cell death protein 1; irAEs: CTLA-4 immune-related adverse effects.		

## 2 ICIs的中枢神经系统不良反应

接受ICIs治疗的患者科出现非特异的神经系统症状，包括头晕、头痛、嗜睡、虚弱、精神萎靡、迟钝等反应，无局灶神经系统体征。对此类现象，在除外神经系统局部病变后给予休息和对症治疗，维持水电解质平衡。

### 2.1 垂体炎

垂体炎的临床表现可引起神经系统症状，特别是头痛、疲劳和虚弱。高达10%的伊普利姆单抗患者发生垂体炎。抗PD-1/PD-L1很少涉及。通常，垂体炎在治疗开始后2个月-3个月出现^[6]^。诊断检查包括内分泌轴（促肾上腺皮质激素、皮质醇、促甲状腺激素、F-T4、促黄体激素、促卵泡激素、睾酮/雌激素、电解质）相关的血清学检查。头核磁共振成像（magnetic resonance imaging, MRI）可显示腺体增大，内分泌改变时需排除其他可能的鉴别诊断，如肿瘤转移等。治疗方面，目前不推荐使用大剂量类固醇（除非是有明显症状的患者）治疗，因为这种治疗不会改善预后。大多数情况下，垂体的破坏是不可逆的，需要长期的激素替代治疗。激素替代治疗开始后可以恢复ICIs治疗。

### 2.2 免疫介导脑炎

ICIs所致的免疫介导脑炎具有症状的多样性和非典型性，诊断较困难，目前已有报道多为病例报道。根据Larkin等近期的研究，免疫介导脑炎多出现在ICIs治疗后的55 d（18 d-297 d）。约0.2%接受PD-1治疗的患者出现免疫介导的脑炎，其中有边缘叶脑炎、脑干脑炎、坏死性脑炎的报道^[7-9]^。免疫介导脑炎的临床表现缺乏特异性，主要以头痛、发热、精神错乱、记忆力障碍、嗜睡、幻觉、癫痫发作、颈强、精神状态下降、注意力受损和定向障碍等脑病症状为主要症状^[10-12]^，一旦出现相关症状，需评估头颅影像学检查MRI及腰穿脑脊液检查。MRI可表现为边缘系统弥散受限^[9]^，亦可表现为大片病灶伴轻度强化^[8, 10]^。腰穿脑脊液检查可提示细胞数及蛋白定量升高^[6]^，细胞数增高以淋巴细胞增多为主，亦可有中性粒细胞增多，中性粒细胞的出现多提示病灶内存在坏死过程；此外，脑脊液IgG水平增高，从侧面提示ICIs对于B细胞功能和分化产生的影响^[13]^。病理方面，多数病例缺乏病理检查结果，根据已有病例报道，irAEs所导致的脑炎在病理上可表现为广泛的脱髓鞘改变、水肿及坏死改变或血管周围及脑实质当中的大量淋巴细胞浸润^[6-8]^。

### 2.3 无菌性脑膜炎

无菌性脑膜炎是一种罕见的副作用，在易普利姆单抗的应用在曾有报道，它通常在ICIs应用后的第1周-第7周间发生。主要症状包括颈部僵硬、发烧和头痛。脑脊液呈无菌液体，以淋巴细胞为主。MRI可见脑膜强化。类固醇治疗通常有效^[14]^。

### 2.4 其他

多发性硬化等炎性脱髓鞘疾病，包括视神经炎、横断性脊髓炎和急性肿瘤性脱髓鞘病变，已在易普利姆单抗治疗期间进行了相应报道。Abdallah等^[15]^报道了1例应用易普利姆单抗后出现横贯性脊髓炎的患者，该患者临床表现以截瘫、尿潴留及下肢感觉障碍为主要表现。全脊髓增强MRI提示T2相斑块状高强化出现在颈髓、胸髓、脊髓圆锥、马尾、骶骨神经根。腰穿脑脊液提示淋巴细胞升高为主的白细胞升高，蛋白质310 mg/dL，葡萄糖27 mg/dL。患者的腰髓硬膜活检病理提示坏死伴组织细胞以及淋巴细胞在血管周围的大量浸润及聚集，无血管壁损伤，无血栓形成。

## 3 ICIs的周围神经系统不良反应

### 3.1 周围神经病

ICIs诱导的周围神经病发生在 < 1%的患者当中，是一种罕见的并发症。Supakornnumporn等^[23]^描述了5例接受ICIs治疗后出现格林-巴利综合征（Guillain-Barre syndrome, GBS）的患者。80%患者在第3个治疗周期出现相应临床表现，主要包括感觉丧失、轻瘫、虚弱、感觉异常、麻木、吞咽困难等。患者脑脊液提示蛋白细胞分离，肌电图提示多发性周围神经脱髓鞘。治疗包括免疫球蛋白治疗、糖皮质激素治疗、免疫球蛋白及糖皮质激素联合治疗、他克莫司治疗、血浆置换治疗等。预后方面，40%患者治疗后死亡，40%患者症状明显好转，20%患者症状维持。

此外，共有3例患者^[24-26]^报道了ICIs治疗后出现面神经麻痹的报道，患者的临床表现主要以典型的周围性面神经麻痹为主，可伴有弥漫性丘疹，头颅MRI检查及脑脊液检查多无明显异常。治疗方面，糖皮质激素治疗有效。

### 3.2 重症肌无力

目前普遍认为，ICIs可导致潜在的自身免疫系统紊乱，目前认为，易普利姆单抗可通过诱导T细胞，介导产生乙酰胆碱受体抗体，以致重症肌无力的发生或恶化。Makarious等^[22]^报告了23例ICI相关的重症肌无力，其中70%为新发病例（13例接受抗PD-1、4例抗CTLA-4和3例联合治疗）。在一大组接受纳武利尤单抗治疗的患者中，重症肌无力的发病率为0.12%。临床表现包括上睑下垂、复视、肌肉无力、呼吸困难和吞咽困难。乙酰胆碱受体抗体在59%的患者中呈阳性。9例同时出现肌炎。多数患者在治疗开始后7周-11周开始出现症状。约1/3的患者在接受积极的类固醇、静脉注射免疫球蛋白（intravenous immune globulin, IVIG）、血浆置换等积极治疗后死亡。

### 3.3 炎性肌病

肌病似乎是抗PD-1/PD-L1最常见的神经系统irAEs，而抗CTLA-4的发病率较低。最常见的类型是坏死性自身免疫性肌炎、皮肌炎和多发性肌炎^[16-19]^。另外还有眼眶肌炎、嗜酸性筋膜炎等较罕见肌病相关的病例报告^[20, 21]^。常见症状包括肌肉疼痛、近端肢体无力、说话困难/吞咽困难、上睑下垂或动眼肌无力。有研究^[20]^描述了19例ICI诱导的肌炎，其中32%出现相关心肌炎，5%伴有重症肌无力，所有患者至少接受抗PD-1治疗。一些患者出现呼吸窘迫，与膈肌受累有关。实验室检查经常显示肌酸激酶升高。电生理检查显示肌源性损害。肌肉活检可见坏死的肌纤维和炎症变化^[20]^。大剂量皮质类固醇和ICIs停药治疗通常可以改善症状。大多数患者完全康复。

## 4 ICIs的神经系统irAEs诊断及相应处理

ICIs的神经系统irAEs的诊断流程见图 1。对于接受ICIs治疗后出现新发的中度至重度神经系统体征或症状的患者，根据临床表现，可完善头MRI、腰穿脑脊液检查、肌电图检查等，重点需排除其他原因，如血管病、进行性肿瘤疾病（脑转移、瘦素瘤病或脊髓压迫）、感染、副肿瘤综合征和毒性/代谢物因素的干扰后方可诊断ICIs的神经系统irAEs^[18]^。快速诊断和治疗是至关重要的，因为神经系统irAEs均可导致严重的后遗症或死亡。对于接受ICIs治疗的患者，一个多学科团队应参与管理决策，因为一方面，停止肿瘤治疗可能会降低药物的治疗效率效率；而另一方面，严重的神经系统irAEs会缩短患者的生存期。在必要的情况下应完善腰椎穿刺及头颅MRI检查，进一步明确病因，尽早开展免疫治疗^[3]^。ICIs的中枢神经系统及周围神经系统irAEs的鉴别诊断思路及相应辅助检查、临床处理如下。

**1:**
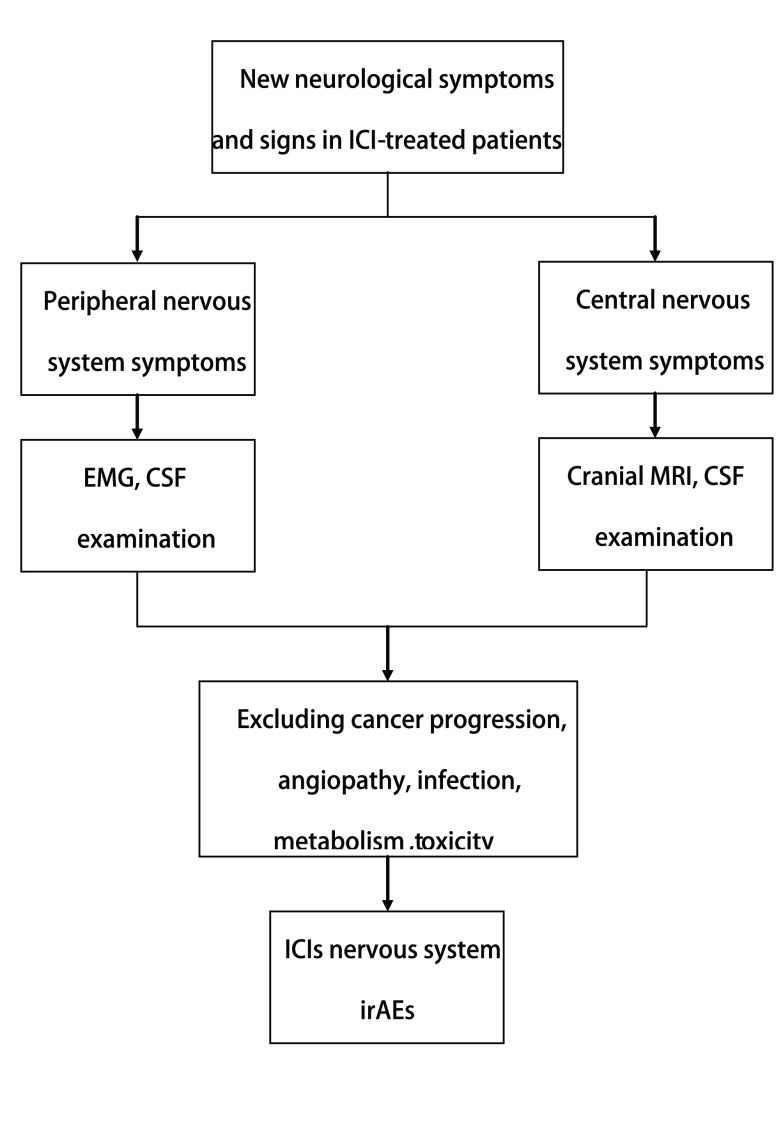
ICIs的神经系统irAEs的诊断流程 Diagnostic flow-chart of ICIs'nervous system irAEs

### 4.1 中枢神经系统ICIs的irAEs的诊疗流程

#### 4.1.1 脑炎

鉴别诊断方面，需与感染性疾病、代谢性疾病、脑转移、脑血管病如脑出血、脑梗死及肿瘤软脑膜转移鉴别，辅助检查方面，血清学检查推荐完成电解质、血糖、总蛋白、血清蛋白电泳及病毒学血清检查，完善头+脊髓MRI，完善腰穿脑脊液检查，检查项目包括白细胞计数、蛋白、糖、氯化物水平、HSV及其他病毒定性以及脑脊液细胞学。疾病处理需根据严重程度分级进行评估，严重程度分级可分为G1级-G4级，G1级是指症状较轻，不限制日常生活，G2级意指新发中度或严重神经系统体征或症状，G3级-G4级指脑炎伴精神行为异常。其中，G1级建议维持ICI治疗，开始诊断流程，如无好转或症状恶化永久停用ICI；G2级可暂维持ICI治疗，密切监测症状及体征，除外病毒或细菌感染（除外感染前可经验性应用抗病毒药物或抗生素）；G3级-G4级患者应永久停用ICI，除外感染后大剂量糖皮质激素（0.5 mg/kg/d-1 mg/kg/d）。

#### 4.1.2 脑膜炎

鉴别诊断方面，需与感染、代谢、肿瘤脑膜播散及肿瘤转移相鉴别。检查方面，建议完善头+脊髓MRI及腰穿脑脊液检查，明确脑脊液白细胞计数，蛋白、糖、氯化物水平，HSV及其他病毒定性，细胞学。治疗方面，除外细菌或病毒感染后应用大剂量糖皮质激素。

#### 4.1.3 横贯性脊髓炎

需与脊髓转移、脊髓压迫、感染性疾病相鉴别，建议完善血清维生素B12、促甲状腺激素（thyrotropin, TSH）、人体免疫缺损病毒（human immunodeficiency virus, HIV）、梅毒、ANA、抗Ro和抗La抗体、AQP4抗体检查，完善头+脊髓MRI，评估腰穿脑脊液白细胞计数、蛋白、糖、氯化物水平、HSV及其他病毒定性、细胞学。治疗方面，可予大剂量糖皮质激素治疗，如激素治疗无效，可考虑IVIG或血浆置换。

### 4.2 周围神经系统ICIs的irAEs的诊疗流程

#### 4.2.1 多发单神经病

鉴别诊断方面，需与代谢、中毒（化疗、维生素缺乏）鉴别，如有颅神经受累，需除外脑膜转移。辅助检查方面，建议完善血清电解质、维生素B12、肌电图检查（electromyography, EMG）检查，评估腰穿脑脊液蛋白、细胞，并进一步完善头MRI。疾病处理需根据严重程度分级进行评估，严重程度分级可分为G1级-G4级，G1级是指症状较轻，不限制日常生活，G2级意指轻度症状，影响日常生活（任意颅神经受累归为G2），G3级意指严重限制日常生活或呼吸受累。G1级建议低剂量维持ICI治疗，观察临床症状变化；G2级建议暂停ICI治疗，监测症状变化或应用糖皮质激素0.5 mg/kg-1 mg/kg，并予对症治疗；G3级建议住院治疗，暂停ICI治疗，并应用大剂量糖皮质激素2 mg/kg。

#### 4.2.2 格林巴利综合征

鉴别诊断方面，需与脊髓压迫、感染性疾病（莱姆、HIV、HSV、HZV）、代谢性疾病、药物副作用、慢性炎性脱髓鞘性多发性神经根神经病（chronic inflammatory demyelinating polyradiculoneuropathy, CIDP）及血管炎进行鉴别，可完善EMG及腰穿脑脊液检查，明确有无蛋白细胞分离，警惕呼吸肌受累，完善血气及肺功能检查，必要时可完善GQ1b抗体检查。治疗方面，首选大剂量糖皮质激素治疗，警惕治疗初期症状恶化；如无改善，可选用血浆置换或IVIG；自主神经功能障碍或呼吸功能障碍的程度决定了是否需ICU进一步治疗。

#### 4.2.3 重症肌无力

鉴别诊断方面，需与代谢性肌炎、毒物诱导的肌无力综合征及多发性肌炎项鉴别，建议完善AchR-Ab、或MUSK-Ab，电生理检查方面完善RNS和单纤维肌电图。治疗方面，停用ICI治疗，建议予大剂量糖皮质激素治疗，并予嗅比斯的明对症治疗；初始治疗无效，可采用IVIG或血浆置换。

#### 4.2.4 肌炎

需与糖皮质激素肌炎、药物相关肌炎鉴别，完善血清CK检查，电生理方面可完善EMG，必要时可行肌肉活检。治疗方面，予大剂量糖皮质激素治疗，如有呼吸肌受累需密切监测血氧，警惕可能的心肌受累。

## 5 ICIs的神经系统irAEs的预后

预后方面，有研究^[27]^全面检索了2016年1月4日-2016年7月3日Bristol-Myers Squibb安全数据库内关于ICIs应用的包括临床试验、病例报告等文献资料，共确定了28例与ICI单药治疗或联合治疗相关的脑炎病例，其中单药和联合治疗组的脑炎死亡率分别为1/19（5%）和1/9（11%），说明联合治疗出现脑炎的死亡率显著高于单药治疗组。ICI在恶性肿瘤治疗中的应用给肿瘤科医师和患者带来了新的希望。ICIs治疗后，神经系统irAEs一旦出现，如果治疗不当，可能危及患者生命，早期识别、早期治疗至关重要。ICIs的神经系统irAEs多为急性或亚急性起病，可能在ICIs治疗期间或之后的任何时间发生。预防或减少神经系统irAEs仍然是一个关键的挑战。然而，目前的共识指导方针仍然基于经验性数据。前瞻性临床试验将成为神经系统irAEs未来研究的关键。
